# The many faces of gastrointestinal dysfunction in stiff person syndrome spectrum disorders

**DOI:** 10.3389/fneur.2023.1273256

**Published:** 2023-10-06

**Authors:** Jacqueline Koshorek, Yujie Wang, Daniela Pimentel Maldonado, Maria I. Reyes-Mantilla, Danielle Obando, Alexandra Balshi, Michael Comisac, Pankaj Jay Pasricha, Scott D. Newsome

**Affiliations:** ^1^Department of Neurology, Johns Hopkins University School of Medicine, Baltimore, MD, United States; ^2^Department of Neurology, University of Washington School of Medicine, Seattle, WA, United States; ^3^Department of Gastroenterology, Johns Hopkins School of Medicine, Baltimore, MD, United States; ^4^Mayo Clinic in Arizona Department of Medicine, Scottsdale, AZ, United States

**Keywords:** stiff person spectrum disorder, GAD65 antibodies, GI dysmotility, gastrointestinal dysfunction, stiff person syndrome

## Abstract

**Introduction:**

The effect of stiff person syndrome spectrum disorders (SPSD) on the gastrointestinal tract (GIT) is unknown. This case series aims to characterize the prevalence and types of GI dysfunction in individuals with SPSD.

**Methods:**

A retrospective chart review included individuals diagnosed with SPSD with descriptors of GI symptoms in their medical records. SPSD phenotypes, type of motility test performed, and dysmotility pattern (upper, lower, or diffuse) were assessed. Descriptive statistics and univariate chi-square analyses were utilized.

**Results:**

Of 240 individuals with SPSD, 32% reported GI symptoms, most were female (83.1%), and white (74%), with a median age at time of GI symptom onset of 50 ± 13 years. Most common symptoms reported were dysphagia (45%), constipation (40%), and nausea/vomiting (23%). Most individuals had classic SPS (47%) followed by SPS-plus (29%) and 82.9% were positive for serum antiGAD65 antibodies. Of 36 patients that underwent at least one GI motility test, 26 had evidence of upper, lower, or diffuse GI dysmotility (44.4%, 17%, and 4%, respectively). The group who did not undergo testing had a higher proportion of patients with DM.

**Discussion:**

There is a high prevalence of GI symptoms and transit abnormalities in patients with SPSD. Future prospective, longitudinal studies are warranted to further assess GI symptoms in the context of SPSD and to determine if individuals with GI symptoms differ in prognosis or treatment response from those without GI symptoms. In the meantime, there should be a low threshold for motility testing in patients with SPSD.

## Introduction

Stiff person syndrome spectrum disorders (SPSD) are rare neuroimmunological disorders that present with a wide spectrum of clinical features and are associated with significant morbidity (1, 2). SPSD are most commonly associated with elevated antibodies directed against glutamic acid decarboxylase 65 (GAD65), a rate-limiting enzyme responsible for the synthesis of gamma-aminobutyric acid (GABA) – the main inhibitory neurotransmitter of the central nervous system (CNS) (3, 4). Although an association of SPSD with diabetes mellitus (DM), pernicious anemia, and thyroid disease is present in the literature, little is known about the effect of SPSD on other organ systems such as the gastrointestinal tract (GIT).

Current literature is limited regarding the prevalence and type of GI symptoms in SPSD. Given that there is a known physiological association of GABA activity and the GI system (5, 6), further characterization of GI dysfunction in SPSD is warranted and increased awareness is necessary to improve management in this population. This case series aims to characterize the prevalence and types of GI dysfunction in individuals with SPSD.

## Methods

A retrospective chart review was performed assessing SPSD patients seen at Johns Hopkins from 1997–2021 as part of a Johns Hopkins Institutional Review Board (IRB) approved ongoing, longitudinal observational study. Patients were included if they had a definitive or probable diagnosis of SPSD and had descriptors of GI symptoms in their medical records. Early on GI symptoms were collected based on self-report, but once it was noted that GI symptoms appeared common, we included questions around GI symptoms as part of our standard clinical SPSD questionnaire which is asked to patients during their clinic encounters. Patients were excluded if GI symptoms were not reported. SPSD phenotypes were classified as classic (torso and limb involvement), SPS-plus (classic features with brainstem/cerebellar involvement), cerebellar (without classic features), progressive encephalomyelitis with rigidity and myoclonus (PERM) and possible SPSD. Details of GI testing were manually extracted for review. Motility testing consisted of nuclear medicine procedures (gastric emptying, small bowel follow-through, colonic transit), manometry procedures (anorectal, esophageal), and/or fluoroscopy procedures (esophagram, modified barium swallow). Results were further categorized into dysmotility pattern; upper (impaired swallow/aspiration, delayed esophageal, gastric, and/or small bowel transit, esophageal spasm), lower (delayed colonic transit, anal and/or rectal spasm) or diffuse (at least one upper GI abnormality and at least one lower GI abnormality). Descriptive statistics are reported. Additionally, univariate chi-square analyses were used to assess differences between those who did and did not complete motility testing, and between SPSD phenotype and dysmotility location. In the latter analysis, possible SPSD was excluded and classic SPSD was compared to those with non-classic SPSD (SPS-plus, PERM, cerebellar).

## Results

Of 240 individuals diagnosed with SPSD, a total of 77 (32%) reported GI symptoms. Those reporting symptoms were mostly female (83.1%), and white (74%), with a median age at time of GI symptom onset of 50 ± 13 years. All but two patients were taking benzodiazepines or antispasmodics when GI symptoms were reported. Most common symptoms reported were dysphagia (45%), constipation (40%), and nausea/vomiting (23%). Most individuals had classic SPS (47%) followed by SPS-plus (29%) (Table 1) and 82.9% were positive for serum antiGAD65 antibodies. One individual tested low-positive for both ganglionic acetylcholine receptor antibodies and antiGAD65 antibodies; in the presence of intravenous immunoglobulin. Three individuals with classic SPS had glycine receptor antibodies. The individual with paraneoplastic-related SPSD had squamous cell carcinoma of the lung and was positive only for antineuronal nuclear antibody 3 (ANNA3).

**Table 1:** Demographics and characteristics of SPSD patients with GI symptoms.

Characteristic	Total population	GI motility test absent	GI motility test present	*p* value
Age (mean ± SD)	49.88 (12.9)	50.41 (13.5)	49.28 (12.2)	0.7
Sex [male, *n* (%)]	13 (16.9)	6 (14.6)	7 (19.4)	0.8
Race [*n* (%)]				
Black	16 (20.8)	11 (26.8)	5 (13.9)	0.23
White	57 (74.0)	29 (70.7)	3 (8.3)	
Other	4 (5.2)	1 (2.4)	28 (77.8)	
SPSD duration, yrs. (mean ± SD)	8.44 (6.7)	8.88 (7.0)	7.94 (6.4)	0.55
SPSD phenotype (*n*, %)				
Classic	36 (46.8)	20 (48.8)	16 (44.4)	0.13
SPS plus	22 (28.6)	14 (34.1)	7 (19.4)	
Cerebellar	2 (2.6)	2 (4.9)	0 (0.0)	
Possible SPS	11 (14.3)	3 (7.3)	8 (22.2)	
PERM	6 (7.8)	2 (4.9)	4 (11.1)	
GI symptoms (*n*, %)				
Dysphagia	34 (44.2)	14 (34.1)	20 (55.6)	0.1
Constipation	30 (39.0)	13 (31.7)	17 (47.2)	0.25
Nausea/vomiting	14 (18.2)	8 (19.5)	6(16.7)	0.98
Abdominal stiffness/spasms (*n*, %)	36 (46.8)	18 (43.9)	18 (50.0)	0.83
Torso stiffness/spasms (*n*, %)	42 (54.5)	23 (56.1)	19 (52.8)	0.95
Positive GAD65 antibodies (*n*, %)	63 (82.9)	35 (85.4)	28 (80.0)	0.754
MRS (mean ± SD)	2.71 (0.82)	2.73 (0.74)	2.69 (0.92)	0.845
Comorbid diabetes (*n*, %)	18 (23.4)	14 (34.1)	4 (11.1)	**0.035**
Comorbid thyroid (*n*, %)	21 (27.3)	13 (31.7)	8 (22.2)	0.499
GI involvement (*n*, %)				
Upper GI^†^			16 (44.4)	
Lower GI^‡^			6 (16.7)	
Diffuse^*^			4 (11.1)	

Cohort Characteristics of the total population and further categorized as those with and without gastrointestinal (GI) motility testing. *p* values were obtained via univariate chi-square analysis utilizing R studio. There were no significant differences in those who did or did not complete motility testing, other than a history of diabetes mellitus being present in a higher number of individuals who did not undergo motility testing. ^†^Upper GI dysmotility: presence of delayed esophageal, gastric, small bowel transit and/or esophageal spasm. ^‡^Lower GI dysmotility: presence of delayed colonic transit and/or anal and/or rectal spasm. ^*^Diffuse GI dysmotility; presence of at least one upper GI abnormality and at least one lower GI abnormality. SD: standard deviation, SPSD: Stiff person syndrome spectrum disorder, GAD: Glutamic acid decarboxylase, MRS: modified Rankin scale, PERM: progressive encephalomyelitis with rigidity and myoclonus.Bold values indicate statistical significance based on *p* value < 0.05.

Almost half of patients (*n* = 36, 47%) underwent at least one GI motility test with dysphagia (56%) and constipation (47%) being the main reasons for diagnostic referral. Twenty-six patients had evidence of upper GI, lower GI, or diffuse GI dysmotility (44.4%, 17%, and 4%, respectively). Obvious transit abnormalities were not detected in approximately 30% of patients. Compared to the group who underwent motility testing, the group who did not undergo testing had a higher proportion of patients with DM (Table 1).

## Discussion

The findings of this study suggest that GI symptoms commonly occur in SPSD and motility testing in this population reveals a spectrum of GIT involvement. The high prevalence of GI symptoms and transit abnormalities in patients with SPSD suggest that gut motility is vulnerable to the same insults as the skeletal neuromuscular system in this condition. Whether this reflects a central or peripheral process is unclear as of yet, although it should be noted that GABA and GABA receptors are highly expressed in the gut and enteric neurons and dysregulation may contribute to symptoms (5, 7).

In individuals predisposed to gut dysmotility, the present study findings could have potential implications in prescribing practices for management of SPSD. Benzodiazepines and/or antispasmodic agents are used as first-line treatment for SPSD. Although these medications potentiate GABA receptors in the CNS which can inhibit or stimulate GABA receptors in the gut based on receptor and location (5), little is known regarding their effect on gut transit in humans (8). In this study, two individuals (non-diabetic) were not on these medications at the report of GI symptoms/motility testing excluding them as a contributor. Despite known GI effects including nausea, vomiting, diarrhea and constipation (9, 10), the role of benzodiazepines and GABA-mediated antispasmodics as possible contributors in SPSD-related gut dysmotility remains unclear.

Although this study represents the largest cohort of SPSD patients with descriptions of GI dysfunction to date, it is limited by the retrospective nature and small sample size. The prevalence of GI symptoms in SPSD could be underestimated given the nonsystematic collection of GI symptoms in the early phase of this study. Similarly, incorporation bias may have led to an increased association between motility testing and prediction of GI dysmotility. Lastly, there was a disproportionate number of individuals who had a history of DM that did not undergo motility testing given the assumption that DM was solely responsible for the GI symptoms/dysfunction. It is unknown at this time whether there is a higher incidence of GI symptoms/dysmotility in those with SPSD alone compared to those with SPSD and DM (or vice versa). It would be important to equally assess those who have comorbid DM and SPSD with motility testing given DM itself could contribute to dysmotility.

Future prospective, longitudinal studies are warranted to further assess GI symptoms in the context of SPSD and to determine if individuals with GI symptoms differ in prognosis or treatment response from those without GI symptoms. In the meantime, it would be prudent to have a low threshold for motility testing in patients with SPSD especially in those experiencing dysphagia and/or constipation.

## Data availability statement

The raw data supporting the conclusions of this article will be made available by the authors, without undue reservation.

## Ethics statement

The studies involving humans were approved by Johns Hopkins Medicine Office of Human Subjects Research Institutional Review Board. The studies were conducted in accordance with the local legislation and institutional requirements. The participants provided their written informed consent to participate in this study.

## Author contributions

JK: Conceptualization, Data curation, Formal analysis, Investigation, Methodology, Writing – original draft, Writing – review & editing. YW: Investigation, Writing – review & editing. DM: Investigation, Writing – review & editing. MR-M: Writing – review & editing, Investigation. DO: Writing – review & editing, Investigation, Project administration. AB: Project administration, Writing – review & editing, Investigation. MC: Investigation, Project administration, Writing – review & editing. PP: Supervision, Writing – review & editing. SN: Conceptualization, Data curation, Formal analysis, Funding acquisition, Investigation, Methodology, Project administration, Resources, Supervision, Writing – original draft, Writing – review & editing.
